# Antibiotic Resistance of Airborne Viable Bacteria and Size Distribution in Neonatal Intensive Care Units

**DOI:** 10.3390/ijerph16183340

**Published:** 2019-09-10

**Authors:** Wendy Beatriz Morgado-Gamero, Martha Mendoza Hernandez, Margarita Castillo Ramirez, Jhorma Medina-Altahona, Stephanie De La Hoz, Heidy Posso Mendoza, Alexander Parody, Elba C. Teixeira, Dayana Milena Agudelo-Castañeda

**Affiliations:** 1 Department of Exact and Natural Sciences, Universidad de la Costa, Calle 58#55-66, Barranquilla 080002, Colombia; 2 Department of Civil and Environmental Engineering, Universidad del Norte, Km 5 Vía Puerto Colombia, Barranquilla 081007, Colombia; 3 Barranquilla Air Quality Monitoring Network, EPA—Barranquilla Verde, Barranquilla 080001, Colombia; 4 Department of Bacteriology, Universidad Metropolitana, Calle 76 No. 42-78, Barranquilla 080020, Colombia; 5 Engineering Faculty, Universidad Libre Barranquilla, Carrera 46 No. 48-170, Barranquilla 080002, Colombia; 6 Postgraduate Program in Remote Sensing, Universidade Federal do Rio Grande do Sul, Av. Bento Gonçalves, 9500, Porto Alegre, RS 91501-970, Brazil

**Keywords:** bioaerosols, neonatal intensive care unit, antibiotic resistance, public health

## Abstract

Despite their significant impact on public health, antibiotic resistance and size distributions of airborne viable bacteria in indoor environments in neonatal intensive care units (NICU) remain understudied. Therefore, the objective of this study was to assess the antibiotic resistance of airborne viable bacteria for different sizes (0.65–7 µm) in private-style and public-style neonatal intensive care units (NICU). Airborne bacteria concentrations were assessed by a six-stage Andersen impactor, operating at 28.3 L/min. Public-style NICU revealed higher concentrations of airborne viable bacteria (53.00 to 214.37 CFU/m^3^) than private-style NICU (151.94–466.43), indicating a possible threat to health. In the public-style NICU, *Staphylococcus* was the highest bacterial genera identified in the present study, were *Staphylococcus saprophyticus* and *Staphylococcus epidermidis* predominated, especially in the second bronchi and alveoli size ranges. *Alloiococcus otitidis*, *Bacillus subtiles*, *Bacillus thuringiensis*, *Kocuria rosea*, and *Pseudomonas pseudoalcaligene*, were identified in the alveoli size range. In NICU#2, eight species were identified in the alveoli size range: *Bacillus cereus*, *Bacillus subtilis*, *Bacillus thuringiensis*, *Eikenella corrodens*, *Pseudomonas aeruginosa*, *Staphylococcus aureus*, *Staphylococcus epidermidis*, and *Streptococcus gordoni.* Multi-drug-resistant organisms (MDROs) were found in both of the NICUs. *Bacillus cereus* strains were resistant to Ampicillin, Cefoxitin, Ceftaroline, and Penicillin G. *Staphylococcus cohnii* ssp. *cohnii* was resistant in parallel to ampicillin and G penicillin. *Staphylococcus saprophyticus* strains were resistant to Ampicillin, Penicillin G, Oxaxilin, and Erythromycin. Results may indicate a potential threat to human health due to the airborne bacteria concentration and their antibiotic resistance ability. The results may provide evidence for the need of interventions to reduce indoor airborne particle concentrations and their transfer to premature infants with underdeveloped immune systems, even though protocols for visitors and cleaning are well-established.

## 1. Introduction

Airborne particles present in the atmosphere for extended periods of time can have profound adverse effects on human health [1]. In recent decades, it has been demonstrated that the performance of the atmosphere acts as a medium of the aerosol-related transmission of infectious diseases and substances harmful to health [2,3,4]. The knowledge on the transportation of bacteria by air currents can aid in the prevention of the airborne spread of pathogens or source identification [5], since bioaerosols released from land and ocean surfaces can be transported over long distances and up to very high altitudes, i.e., between continents and beyond the troposphere [6]. Bioaerosols are airborne particles of a biological origin. This includes fungi, bacteria, viruses and/or pollen, and fragments of the foregoing or their metabolic products [7]. The survival, dispersion, and reproduction of these depend, to a large extent, on the environmental conditions in which they are found, and factors such as temperature, relative humidity, air movement, light, and food sources [8,9]. Currently, the importance of bioaerosols and their impact on human health have gained increasing attention in different sectors of our society, including the health sector [3], since they pose potential exposure risks to patients and health workers [10] for the acquisition of infections [11]. A marked increase of infections—i.e., pneumonia, tuberculosis, gonorrhea, and salmonellosis—have been occurring in which their treatment becomes more difficult due to the loss of efficacy of antibiotics. Consequently, antibiotic resistance may occur when bacteria mutate in response to the use of these drugs.

Several kinds of research have focused on the study of bacterial bioaerosols present in various health institutions, justified by the favorable conditions presented by these environments for the transport and survival of microorganisms in the air [12,13]. Sources of bacterial bioaerosols that cause hospital infections may include patients, environment, and hospital staff [14]. The study of bioaerosols concentration and antibiotics bacterial resistance evaluation plays an important role in the prevention of hospital-acquires infections (HAIs) and can be useful for the design of strategies that protect both employees and patients, taking into account that each of them has different degrees of susceptibility or immunosuppression and may be affected by poor control of these elements [15]. In addition, these studies may provide information for the epidemiological investigation of IIH diseases, their propagation, control of microorganisms in the air, monitoring of biological procedures, and their use as a quality control measure [14]. However, there is a lack of studies of bacterial resistance of bioaerosols, even though it is considered one of the most serious threats to global health since it increases medical costs, prolongs hospital stays, and increases mortality [16].

Consequently, the aim of this study was to assess the antibiotic resistance of airborne viable bacteria for different sizes (0.65–7 µm) in neonatal intensive care units (NICU). The objectives were: (i) to measure airborne bacteria concentrations before and after family visits, (ii) to identify species of bacteria, (iii) to characterize particle number concentrations simultaneously with airborne bacteria, and (v) to identify antibiotic resistance of airborne bacteria. This work will generate fresh insight into the understanding in private and public-style NICU of airborne bacteria particle size distribution, possible association to particle number, effect of visitors, and cleaning activities. The results of this research may be used for exposure and health-risk assessments for infants in NICU environments. Also, they may provide valuable evidence for the need of interventions to reduce indoor airborne particle concentrations and their transfer to premature infants with underdeveloped immune systems, even though protocols for visitors and cleaning are well-established. 

## 2. Materials and Methods 

In this research, the measurement of airborne concentrations was conducted in two steps: aerosol sampling in the neonatal intensive care unit (NICU), and bacteria identification in the laboratory [17].

### 2.1. Neonatal Intensive Care Units (NICU) 

**Figure 1:**
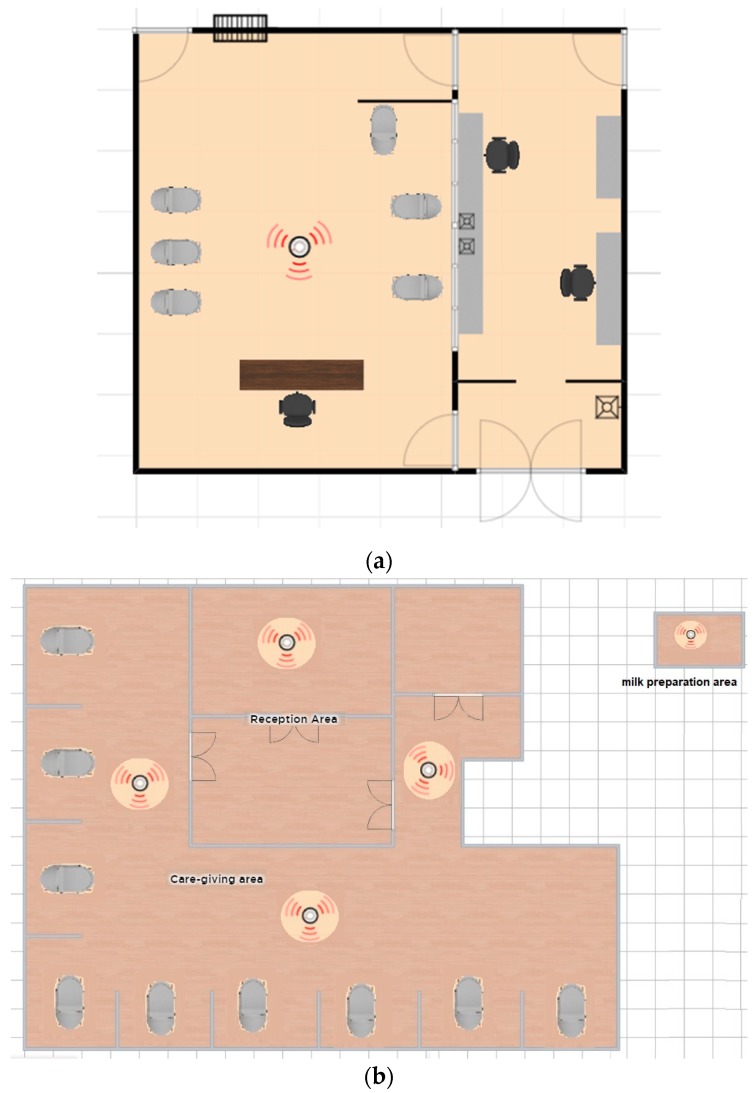
Outline of the neonatal intensive care units (NICU)#1 (**a**) and NICU#2 (**b**).

The field-monitoring portion of the study was conducted at two different NICU of acute care hospitals located in Barranquilla, a city in the northern Caribbean cost of Colombia. Measurements were made in a private-style NICU (1), and a public-style NICU (2). The first NICU, NICU#1, is located in a university hospital, a non-profit institution that was founded to support medicine teaching work. This hospital provides services of Therapeutic Support, Diagnostic Support, Emergencies, Hospitalization, and Outpatient. The second NICU studied, NICU#2, is located in a health center from the Public Hospitals Network. This hospital is enabled for hospitalization, burn care unit, adult intensive care unit, pediatric intensive care unit, and neonatal intensive care units, providing special attention in Cardiology and Hemodynamics. These hospitals provide medical attention to vulnerable groups of people in terms of poverty in Barranquilla, and their care statistics show close to 17,000 patients per year. NICU#1 had been configured to provide a personalized care-giving environment and services for 26 neonatal babies (less than one month). This one is located on the third level of the hospital, the entire floor area of the NICU is 32 m^2^. In this unit, visits are allowed once per day in a specific hour. The physical layout of the NICU#1 can be observed in Figure 1a. The sampler was placed in an equidistant location between the neonatal beds in order to have representative samples of the area. The second NICU studied, NICU#2, is located on the second level of the hospital, and the entire area is 360 m^2^, which is enabled for providing personalized attention to 30 neonatal babies. This unit has a program called kangaroo mom, which means that mothers can visit their babies at any time of the day. Figure 1b shows the five points where the sampler was located.

In both NICU, air conditioning maintenance and cleaning protocols are allowed by National requirements of the unique system of qualification. These requirements indicate that acute care hospitals must have different protocols for general patient rooms and intensive care units, which must include detergent rotation, an air conditioning maintenance program, and superficial microorganism control monitoring. These requirements had been reviewed by the Colombian National Health Ministry through the District Health Secretary of the city, but do not include specific protocols to ensure the air quality of the indoor environment or requirements for monitoring bioaerosols or particle matter samples. Both units are covered with hard tiles which were cleaned approximately every 24 h by using microfiber mops that were used exclusively for this area. All individual baby rooms were cleaned daily and there were no evident signs of dust accumulation. Both units have High Efficiency Particulate Air (HEPA) filters which must be changed every six (6) months and they have a proprietary condenser for the exclusive operation of the area. Five (5) bacteria bioaerosols sampling campaigns were made in NICU#1 and three (3) bacteria bioaerosols and particle number campaigns were done simultaneously in NICU#2.

### 2.2. Airborne Bacteria and Particle Number Samples Collection

**Figure 2:**
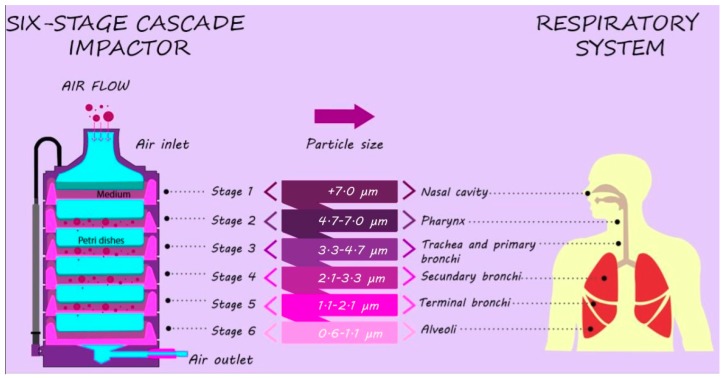
The particle size of the six-stage cascade impact and respiratory system.

Airborne viable bacterial sampling was performed using an Andersen six-stage cascade impactor (Thermo Fisher Scientific©, Walthman, MA, USA) on Petri dishes, containing Plate count Agar of Merck© (Darmstadt, Germany). The optimal sampling time collection used in our research, of 5 min, was found in previous research [18,19]. Three consecutive replicates were taken for each single sampling time. The sampler worked at a constant flow rate of 28.3 L/min and was sterilized before and after use by using 75% ethanol. All inside surfaces were maintained in sterile conditions until sampling, and blank control was done. Plate count agar Petri dishes were left unsampled in each of the stages of the study: media preparation, transport of Petri dishes, sampling, transport of the samples to the laboratory, and incubation. The sampler collects particles in six stages according to their aerodynamic diameter: >7.0 μm, 4.7–7.0 μm, 3.3–4.7 μm, 2.1–3.3 μm, 1.1–2.1 μm, and 0.65–1.1 μm, corresponding to particles size that may penetrate the nasal cavity, pharynx, trachea and primary bronchi, secondary bronchi, terminal bronchi, and alveoli, respectively (Figure 2) [19,20,21,22,23]. Simultaneously, environmental indoor conditions data were recorded by a Kestrel anemometer Model 4500, including information of ambient temperature and relative humidity, to validate that environmental conditions had been controlled according to the health safety protocols established by the Hospital.

Airborne viable bacteria were sampled in two neonatal intensive care units (NICU) in two different acute care hospitals. Sampling in NICU#1 was done during 5 sampling campaigns, before and after the visiting time, therefore 196 samples were collected. Sampling in NICU#2 consisted of 3 campaigns, before and after cleaning activities. 

Particle number concentrations in NICU#2 in five size ranges were measured: channel 1 (0.3–0.5 μm), channel 2 (0.5–1 μm), channel 3 (1–2.5 μm), channel 4 (2.5–5 μm), and channel 5 (2–10 μm), using a particle counter from Hal Technologies (HAL-HPC601). The particle counter simultaneously measures 6 user-configurable particle sizes. It is manufactured in the USA and is in compliance with the international standards (JIS B 9925:1997 and ISO21501 and ISO14644-1) and CE certified.

### 2.3. Bioaerosol Cultivation and Concentration Assessment 

Bacteria samples were incubated at 37 °C ± 2 °C for 48 h. The counting of the colony forming units (CFU) per Petri dish was based on typical Gram staining and their macroscopic characteristics (shape, color, texture, and border, among others) [24]. Airborne viable bacteria concentration was determined by dividing the CFU by the sampled air volume, applying Equation (1) expressed in CFU/m^3^.

(1) $$\mathrm{Bioaerosols}\;\mathrm{concentration}\;\left(\frac{\mathrm{CFU}}{m^{3}}\right)=\frac{\left(N\;{}^{\circ}C\times 1000\right)}{F\times t\;}$$

where N °C is the number of colonies per plate, 1000 is a unit conversion factor, F is the flow rate of the six-stage cascade impactor (28.3 L/min), and t is the sampling time (5 min). 

### 2.4. Bacteria Identification and Antibiotic Resistance 

**Figure 3:**
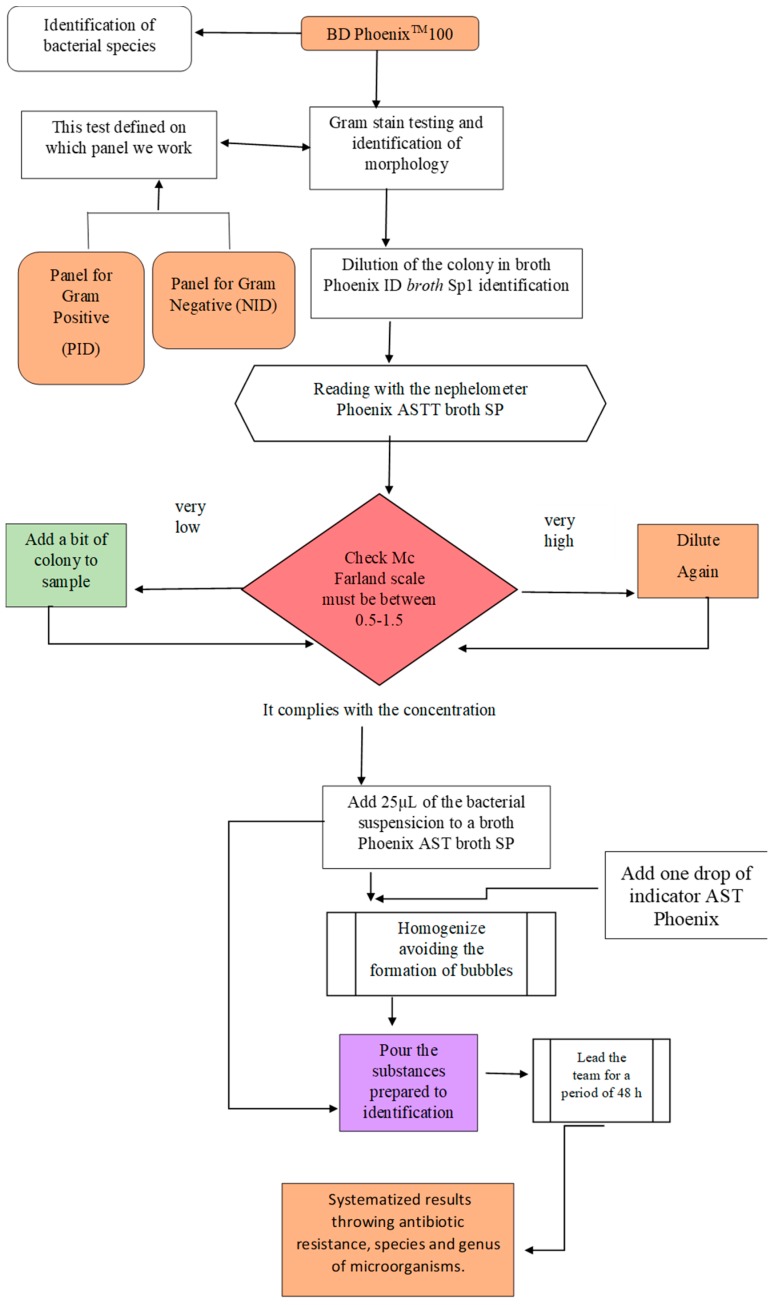
Methodology used for identification with BD Phoenix™.

BD Phoenix™ 100 (Becton Dickinson & Company, Franklin Lakes, NJ, USA) was used to identify the genus, species, and resistance to antibiotics, which performs continuous monitoring for susceptibility and identification tests using two detection methods (Fluorometry and Turbidimetry). The equipment has panels, which are placed in the inoculation station with the holes in the upper part, and takes the isolated colonies that were previously confirmed by means of Gram stain, in order to choose the correct panel. Figure 3 shows the methodology used for identification with BD Phoenix™. The colonies under study in the broth of ID (bacterial identification) were taken to 0.5 of McFarland (acceptable at 0.5–0.6) for the exact volume and if it was added could overflow the panel. Once the inoculum is prepared, the ID tube is vortexed, and its turbidity is measured in the nephelometer. Later, an AST and/or AST-S indicator solution was added to the AST and/or AST-S broth (solutions that help in the identification of bacterial resistance), which goes on one side of the panel and the other panel side will broth the ID (Bacterial Identification) broth. Once it is done it is taken to the equipment, in which it has a time of analysis at 12 h for ID (36 readings) and 16 h for AST (48 readings).

In addition to the identification of the genus and bacterial species, the BD Phoenix™ had determined the resistance or test of susceptibility of the species to the different antibiotics that are usually used. A total of 27 antibiotics were reviewed. The studied antibiotics were Ampicillin, Cefoxitin, Ceftaroline, Penicillin G, Gentamicin, Daptomycin, Linezolid, Vancomycin, Amikacin, Cefepime, Ceftazidime, Ciprofloxacin, Imipenem, Levofloxacin, Meropenem, Piperacillin-Tazobactam, Ampicillin-Sulbactam, Cefazolin, Ceftriaxone, Ertapenem, Oxacillin, Minocillin, Rifampin, Trimetropin-Sulfamethoxazole, Clindamycin, Erythromycin, and Tigecycline.

### 2.5. Data Analysis

Data were systematized in a spreadsheet by sampling campaigns, before or after visiting time, and replicate samples, as independent variables. The data were analyzed using the software Statgraphics Centurion XVI, applying a generalized linear regression model, in order to determine if there was a relationship between the variables measured (before and after visiting time) and the concentrations obtained from bacterial bioaerosols with 95% of confidence (*p* < 0.05). In addition, a statistical analysis was carried out using a Multifactorial Analysis of Variance (ANOVA) that allowed us to establish whether there were significant differences between independent factors or variables.

## 3. Results

### 3.1. Size Distribution and Bacteria Bioaerosols Concentration

No uniform international standard is established for acceptable limits of bioaerosol concentrations. The World Health Organization (WHO) has not yet established a specific guide for concentration levels and current standards based on health risk assessment. However, some private and governmental organizations have established quantitative standards and guidelines for bioaerosols in indoor environments. These standards are based on reference values for bioaerosol concentrations, without taking into account the effects on human health. Since the No-Observed-Adverse-Effect-Level (NOAEL) or Lowest-Observed-Adverse-Effect-Level (LOAEL) depending upon dose-response approach has not been well-established for bioaerosols concentration, health effects in relation to exposure limits on the basis of data from epidemiological and toxicological studies could not be developed to date [25]. 

Table 1 presents a comparison of data of the present study with bioaerosols guidelines by governmental and private organizations. For our research, total bacterial concentration ranged between 53.00 and 214.37 CFU/m^3^, with a mean concentration of 110.13 CFU/m^3^ between campaigns for NICU#1. While, for NICU#2, bacterial concentrations ranged between 151.94–466.43 CFU/m^3^, with a mean concentration of 310.37 CFU/m^3^. Although detailed studies of bacterial bioaerosols in ICU settings are scarce in the literature, results of our research are similar to other studies done in public buildings and ICU [10,23,26,27,28]. The different concentrations in total viable bacteria in air between the two NICU studied, demonstrates the possible influence of personnel. As demonstrated in other research, personnel visiting the NICU are sources of bacterial aerosols [10,29]. In general, our results could be classified as intermediate (100–1000 CFU/m^3^) by the American Conference of Governmental Industrial Hygienist. In terms of the Indoor Air Quality Association and the Healthy Buildings International, this result is considered safe when species are not infectious or allergenic. In contrast, according to the Netherlands Research Methods, these values of total bacteria in air could be a possible threat to health. As for the Occupational Safety and Health Administration, these concentrations indicate contamination [10,30,31,32]. A comparison of the results with these guidelines may suggest the need to analyze the influence of occupants, visitors, and human activities, as well as the efficiency of hospital ventilation/filtration systems. As explained above, these two NICU (ICU for babies < 1 month) meet the national requirements of the unique system of qualification [33], the differences reside in the personnel visiting protocols. For the NICU#1, visiting hours are stated and fixed, while for NICU#2 visitors may enter at any time. 

**Figure 4:**
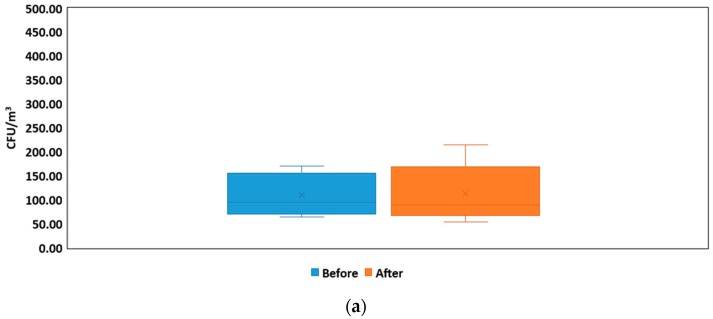

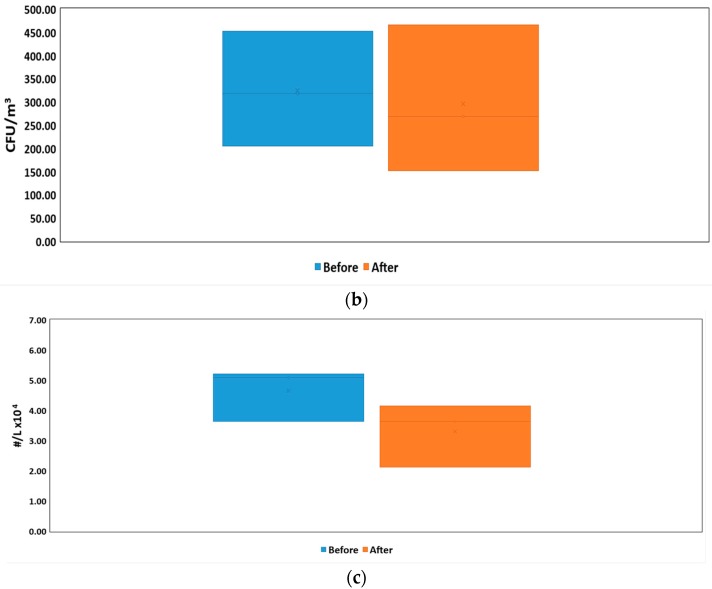
Box plots for the total bacterial bioaerosols concentration (CFU/m^3^) in the NICU#1 before/after visits with median, quartiles, and outliers (**a**) and in the NICU#2 before/after cleaning (**b**,**c**).

The characteristics and concentrations of bacterial bioaerosols might be influenced by many factors, thus the first set of questions aimed to compare the influence of visitors entering the NICU#1. First, total bacterial concentrations were calculated using Equation (1) for the sum of all stages: >7.0 μm, 4.7–7.0 μm, 3.3–4.7 μm, 2.1–3.3 μm, 1.1–2.1 μm, and 0.65–1.1 μm, in order to better compare the two groups (before and after visits). Second, box plots, given in Figure 4, were calculated. Figure 4a illustrates the variation in the NICU#1 before and after visits, showing that bacterial bioaerosol concentrations were slightly higher before visits, although higher concentrations were observed sometimes after visits. Consequently, ANOVA was used to analyze the results before and after the visit time in order to confirm this (or not). None of these differences were statistically significant between the two groups for a *p*-value > 0.05 and a confidence level of 95.0% (Table 2). Third, box plots of airborne bacteria concentration for NICU#2 were calculated in order to analyze if cleaning activities are enough to reduce bacterial bioaerosol concentration (Figure 4b). Figure 4c shows the box plot that illustrates the reduction in particle numbers after cleaning activities. ANOVA (Table 2) results indicated no statistical differences between the two groups (before and after). It is possible to hypothesize that bacterial bioaerosol concentrations did not increase after visits. Also, that cleaning activities did not reduce bioaerosol levels. Moreover, particle number concentration was analyzed, confirming that standardized cleaning activities did not significantly reduce airborne particles. 

**Figure 5:**
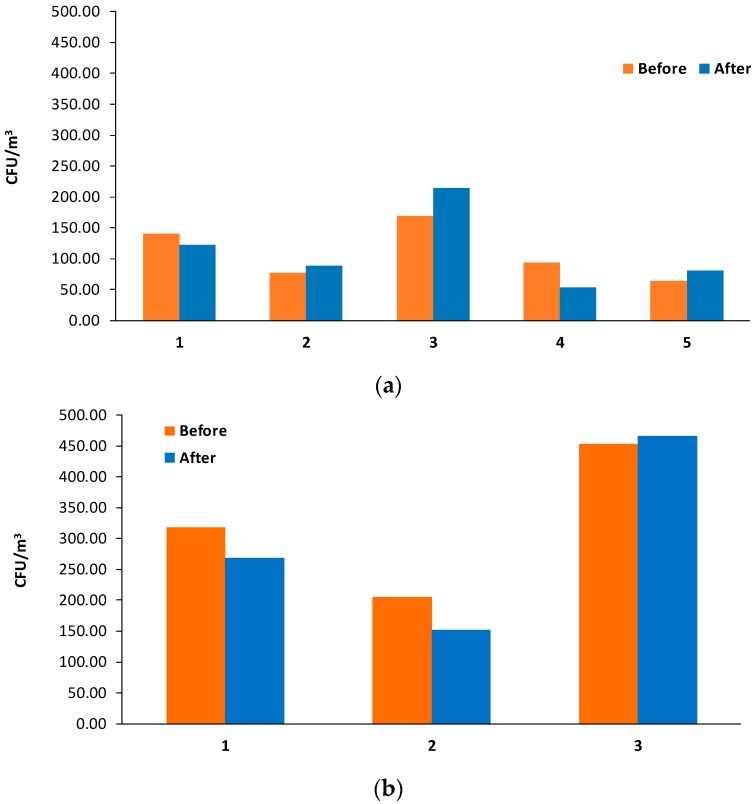
Total bacterial bioaerosols concentration (CFU/m^3^) in the NICU#1 before/after visits (**a**) and before/after cleaning in NICU#2 per campaign (**b**).

Moreover, results were analyzed for each sampling campaign (Figure 5). Figure 5a shows the results of bacterial bioaerosols concentration during the five monitoring campaigns before and after visits, for all six stages (NICU#1). Environmental conditions during the five campaigns presented ranges of temperature and humidity of 27–24 °C and 74%–63.2%, respectively. Each campaign showed different concentrations. The highest values were observed in campaign 3. On the other hand, the minimum total concentration for all stages was obtained in campaign 5 before the visit. Thus, a decreasing tendency in concentrations was observed after campaign 3. These results possibly demonstrate the effect of a cleaning day journey of air conditioning filters after campaign 3. Several studies of microbiological air monitoring, before and after cleaning activities in health institutions, attempted to explain how the activities of general cleaning, air filter maintenance, and the operation of other routine sources may vary bioaerosol concentration in an indoor environment [28,39,40]. 

Campaign results for NICU#2 are shown in Figure 5b. Environmental conditions during the three campaigns presented ranges of temperature and humidity of 23.1–31.7 °C and 56.2%–82.3%, respectively. Results indicate higher values during campaign 3, although no significant differences between concentrations before and after cleaning. In the study carried out by [28], the results demonstrated that the main sources influencing the indoor environment’s air quality in a pediatric intensive care unit include cleaning activities. Therefore, in NICU#2, the effect of cleaning activities was evaluated.

**Figure 6:**
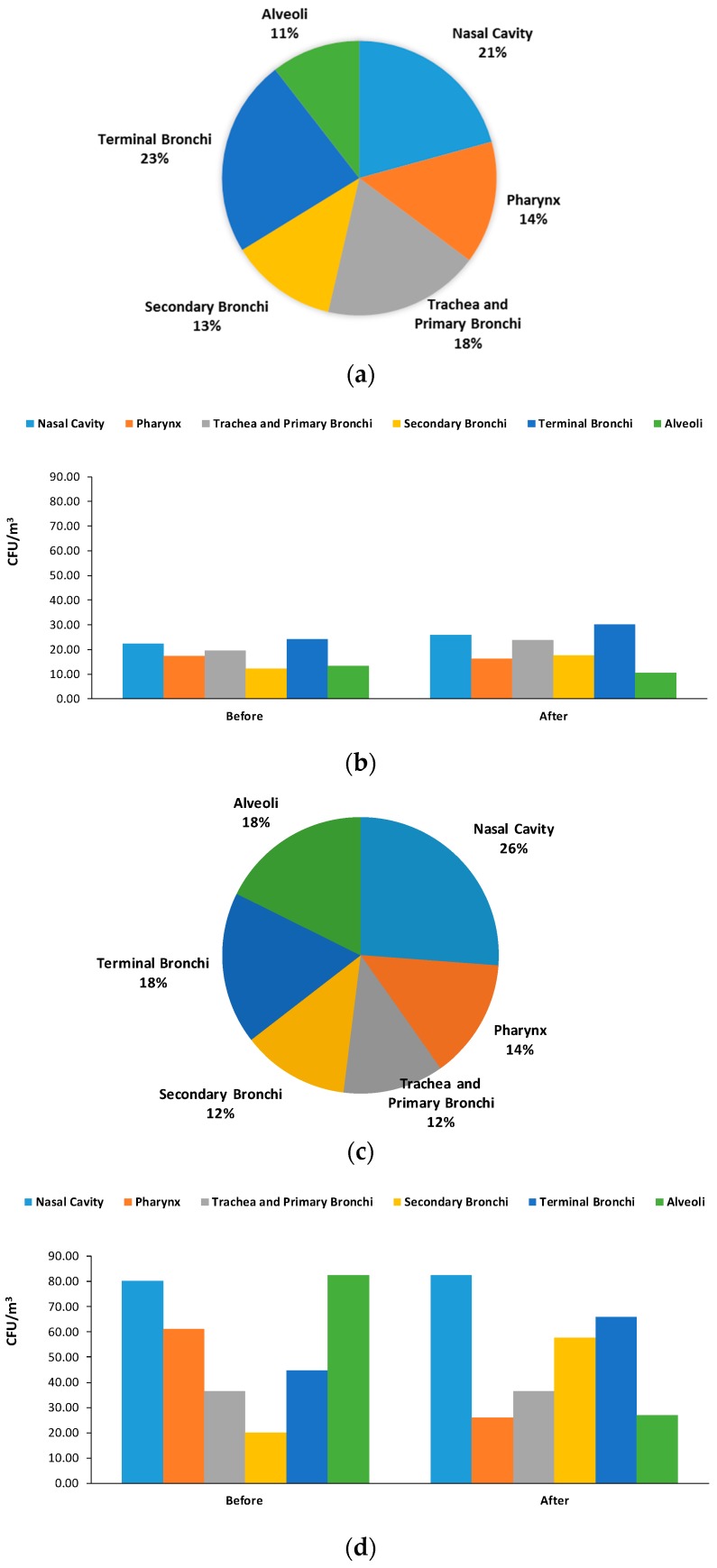
Size distribution of bacterial bioaerosols concentration per stage before/after visit (**a**,**b**), and before/after cleaning (**c**,**d**).

Figure 6 presents the size distribution of bacterial bioaerosols concentration obtained in the NICU. For NICU#1, airborne bacteria concentrations were similarly distributed for all size ranges (Figure 6a). Although, the highest mean concentration was obtained for terminal bronchi and nasal cavity size range. Figure 6b shows a similar distribution, were the highest concentration was in the terminal bronchi stage before and after visits, yielding values of 24.03 and 30.153 CFU/m^3^, respectively. This size range had the highest bacterial bioaerosol concentration in indoor environments, similar to other research [41]. This is a rather significant finding since smaller particles are more likely to penetrate the respiratory system [42]. Overall, these results indicate that approximately 46% of the total viable bacteria collected in the six-stage impactor were in the size range of 0.6–2.1 μm that can penetrate and deposit in the secondary bronchi, terminal bronchi, and alveolus. Therefore, 53% of the bacterial bioaerosols were in the size range of coarse atmospheric particles (>2.1 μm): nasal cavity, pharynx trachea, and primary bronchi [43,44]. For NICU#2, airborne bacteria concentration (Figure 6c) predominated in the nasal cavity size range (26%), terminal bronchi (18%), and alveoli (18%). Values ranged between 20.02–82.45 UFC/m^3^, where the highest concentration corresponded to nasal cavity and alveoli size ranges (Figure 6d). 

It is important to note that, the small size of these particles means that they can enter the lungs easily if inhaled [25]. Bacterial bioaerosol in indoor environments’ variable concentrations and size distribution patterns depend on the microorganism type, local climate, microclimatic factors, level of occupation, type of human activity, type of ventilation, and building maintenance [25,45,46,47]. Most likely, the bacterial bioaerosol concentration in the coarse size range obtained in our study was influenced by the positive correlation between atmospheric particle matter and bacteria size. Bacteria adhere to the particle matter, thus increasing the size of the particle [20,44,48]. On the other hand, bacterial bioaerosol concentration in the fine fractions is due to their greater surface area that allows greater adhesion. This situation, coupled with the fact that indoor environments present a higher concentration of fine particles that may remain suspended, may contribute to this finding [27,44,49,50].

### 3.2. Species Identification and Mean Concentration in Air

Sensitivity of the hospital building environment due to the presence of potential sources of a wide range of airborne microbes, make it a complex environment [51]. Table 3 shows the mean concentration of bacterial bioaerosols per campaign, before and after visits in NICU#1. Gram-positive bacterial bioaerosols had represented 92% of the strains isolated. Furthermore, 12 different identified bacterial strains belonged to six genera: *Alloiococcus*, *Bacillus*, *Kocuria*, *Leifsonia*, *Pseudomonas*, and *Staphylococcus*. Within the *Staphylococcus* genus, three different species were found (*Staphylococcus epidermidis*, *Staphylococcus saprophyticus*, and *Staphylococcus cohnii* ssp.), while for the genus *Bacillus* five different species were found (*Bacillus cereus*, *Bacillus megaterium*, *Bacillus pumilus*, *Bacillus subitilis*, and *Bacillus thuringiensis*). The other genera presented only one species for each one: *Alloiococcus otitidis*, *Kocuria rosea*, *Leifsonia aquatica*, and *Pseudomonas pseudoalcaligene*. 

Moreover, four predominant species were obtained with significant concentrations. The highest average concentration was obtained by the species *S. saprophyticus*, this species had been in all of the sampling campaigns, followed by *S. epidermidis*, which can migrate from the skin along the surface of the device into the body, forming a highly organized bacterial community known as biofilm [52]. It can be transported to the air due to airborne staphylococci that may be shed by carriers and may remain viable for a long time [53]. Similarly, the mean concentration of the species *Kocuria rosea* was 8 and 28 CFU/m^3^ before and after the visit time. *Kocuria* genus are usually considered to be contaminants, but the involvement in human infections has been documented, they can cause major infections, mostly in immunocompromised hosts or patients with serious underlying conditions as neonatal babies [54,55]. Additionally, *Kocuria* is one of the radiation-resistant bacteria [56]. 

Another microorganism with a representative concentration was *Bacillus cereus*, that presented a concentration of 9 and 7 CFU/m^3^ in four campaigns, with the exception of campaign 3 before visits, when it had a concentration of 21 CFU/m^3^, *B. cereus* is able to form endosporeforming rods. It is of concern because humans are contaminated via the spores that remain metabolically inactive in the environment but take the form of vegetative cells when they infect the human body. *B. cereus* as the causative agent of a nosocomial bacteraemia outbreak has been reported in the neonatology hospital ward, emergency department, and intensive care units [57]. 

Regarding the other species identified *Alloiococcus otitidis*, *B. megaterium*, *B. pumilus*, *B. subtilis*, *B. thuringiensis*, *L. aquatica*, *P. pseudoalcaligene*, and *S. cohnii* ssp., all kept low concentrations of 7 CFU/m^3^. As it was mentioned previously, Staphylococcus was the genus with the highest prevalence, probably because this microorganism has a thick cell wall that provides greater tolerance to desiccation and allows it to survive, remaining viable for a longer time [58]. Similarly, *B. pumilus* has been identified as an opportunistic pathogen [59,60], it rarely causes serious infections and has only been described in newborns and immunocompromised individuals [61]. Other species of the Bacillus genus found in the study, such as *B. megaterium*, *B. subtilis*, and *B. thuringiensis*, are common in the soil. The pathogenic potential of these Bacillus is generally described as low or absent [62]. However, they have been increasingly recognized as biological indicators of contamination and may represent a danger to the hospitalized or immunosuppressed patient. Infections or diseases caused by these bacteria are rare and few clinical studies are recognized, some are related to trauma, deep soft tissue infections, and systemic infections [63,64]. The study also reported *Alloiococcus otitidis*, a pathogen associated with otitis media with effusion (OME), a common disease in childhood [65,66]. This microorganism is not considered an opportunistic pathogen or a cause of nosocomial diseases [67]. *Pseudomonas pseudoalcaligene* uniquely identified Gram-negative in our research is shown as a rare opportunistic pathogen associated with intrahospital infections in humans, such as meningitis and pneumonia [68]. It is important make emphasis of the *Leifsonia aquatic* in this NICU, because infections associated with this bacteria are particularly uncommon, nevertheless there had been documented cases of hemodialysis patients involving *L. aquatica* bacteremia [69].

Table 4 shows the mean concentration of bacterial bioaerosols per campaign before and after cleaning in NICU#2. Our research identified some of the species which are considered as aggressive pathogens and highly dangerous for the health of the newborns, considered capable of causing respiratory infections, cases of pneumonia, and infections of the skin and eyes. This was the case with *Staphylococcus aureus*, which is both a prominent cause of nosocomial infections with significant morbidity and mortality, and a commensal with nasal carriage in around 30% of the population [70]. Other species are recognized as pathogenic under certain circumstances, such as the case of *S. epidermidis*, a normal bacterium of human skin [71,72]. However, this microorganism can also emerge as an intrahospital pathogen that infects immunocompromised patients [73,74,75]. Infections caused by *S. epidermidis* are related to the colonization of foreign bodies (intravenous catheters, hemodialysis fistulas, pacemakers, prosthetic joints, valvular grafts and/or prosthetic heart valves) and may eventually produce urinary tract infections, bacteremia, and sepsis [76]. 

In general, NICU#2 presented a higher concentration of bioaerosols in comparison with NICU#1, *B. subitilis* had the highest concentration before and after cleaning protocols, followed by *B. cereus*, this is concerning because of the increasing antibiotic multi-resistant strains that have been reported. On the other hand, *Eikenella corrodens* presented a significant concentration before and after cleaning in NICU#2, and has been reported in head and neck, heart, intraabdominal, and intracranial infections [77]. *Streptococcus gordoni* is a species which had not been found in NICU#1, but had been identified in NICU#2. *S. gordonii* is considered a primary oral colonizer in dental plaque formation, and constitutes the platform on which late pathogenic colonizers operate [78]. 

*Pseudomonas aeruginosa* is also commonly found in moist areas of hospital environments, such as it was found in NICU#2. It can cause opportunistic infection in immunocompromised patients. Preterm neonates are at increased risk of severe or fatal infection with *P. aeruginosa* due to their immature immune systems and the multiple invasive procedures they undergo. There have been several high-profile outbreaks of nosocomial *P. aeruginosa* infection in neonatal intensive care units (NICUs) [79]. 

**Figure 7:**
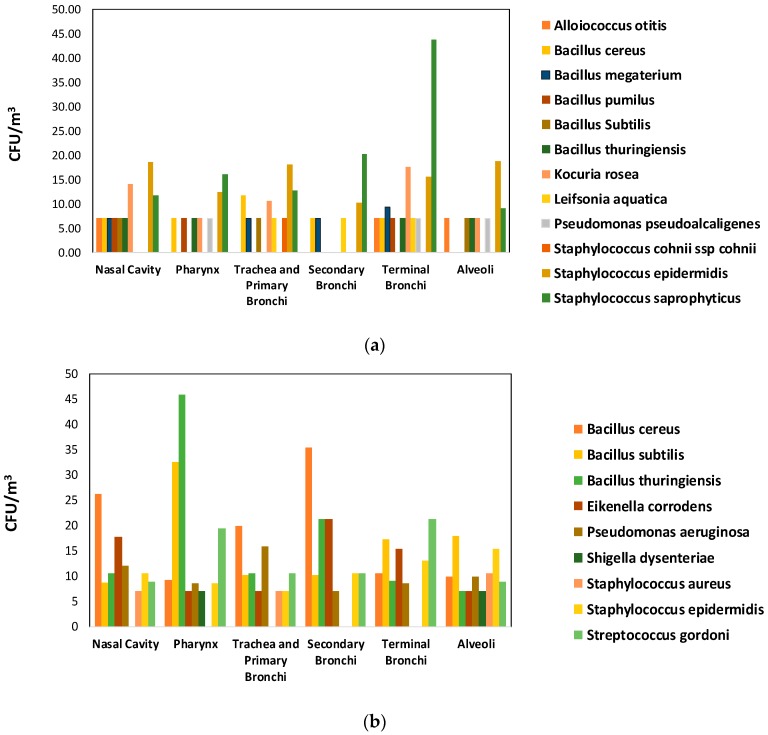
Size distribution of bacterial species concentration. (**a**) NICU#1, (**b**) NICU#2.

In addition, Figure 7 shows species founded in each part of respiratory corresponding to the cascade impactor sampler: nasal cavity (stage 1), pharynx (Stage 2), trachea and primary bronchi (Stage 3), secondary bronchi (Stage 4), Secondary (Stage 5), Alveoli (Stage 6). Figure 7a, shows the species identified in NICU#1, it may be evidenced that species of *Staphylococcus* were presented in all the stages, especially in the last and critical stages, bronchi and alveoli. There is a prevalence of *S. saprophyticus*, considered a primary pathogen at the urinary level, agent of extra-hospital urinary infection in young women and children [71,80]. The infection is acquired in the community; therefore, the microorganism is not considered a hospital-acquired infection (HAI) agent [71]. In contrast, *S. cohnii* ssp. is recognized as a pathogen or opportunist causing HAIs [81]. It is associated with bacteremia, acute cholecystitis, brain abscess, endocarditis, pneumonia, and urinary tract infection [82,83]. On the other hand, *K. rosea* is part of the normal microbiota of the skin, mouth, and oropharynx of humans and other mammals [84]. It is an uncommon pathogen and under certain circumstances causes diseases in immunocompromised patients, so it is considered opportunistic [85]. In the literature, there are only a few medical reports associated with vascular catheters, bacteremia, cholecystitis, and peritonitis in chronically debilitated patients [86]. In the case of *B. cereus*, it is an uncommon but potentially serious pathogen, associated with very low-frequency infections, it produces diseases in an isolated manner and it is mainly recognized as a pathogen with a high mortality rate in neonates [87], capable of causing hemorrhagic meningoencephalitis, respiratory infections, bloodstream infections and affecting the central nervous system of preterm infants [88,89]. Figure 7b, shows that in NICU#2, there is a prevalence of *Staphylococcus* in all the stages, represented by *Staphylococcus aureus* and *Staphylococcus epidermidis*. *S. aureus* is both a prominent cause of nosocomial infections with significant morbidity and mortality, and a commensal with nasal carriage in around 30% of the population. It may be evidenced that in this study, *S. aureus* and *S. epidermidis* were founded in the upper respiratory tract and the lower respiratory track; including they had appeared in Alveoli stage.

Multi-regression statistical analysis was performed for the total concentration data before and after visits. Results showed a significant difference between concentrations for *K. rosea*, *Bacillus Cereus*, *Staphylococcus epidermidis*, *Bacillus megaterium*, and *Staphylococcus saprophyticus* before and after visits. Statistical analyses showed a significant difference for concentrations before and after the visit in all stages. These results are included in the Supplementary information (SI). Thus, even though results did not show differences between total airborne bacterial concentration (as explained above) before and after visits, for bacterial levels for each species, results show the contrary. This indicates that in results for species, visits have an influence. On the contrary, for NICU#2, statistical analysis applied using ANOVA indicated no significant difference between groups before and after cleaning activities. Moreover, correlation between particle number and airborne bacteria concentration did not show any relation by size distribution or species (see SI). 

Coagulase-negative staphylococcus (CONS) is a common cause of nosocomial infections and is the most common bloodstream infection treated in neonatal and pediatric intensive care units and significantly impacts patient mortality and morbidity [90]. *S. saprophyticus* and *S. epidermidis* had a relevant concentration in both NICUs, they were able to be established in all size ranges, subsequently, predominance of these species had been presented in the terminal bronchi and alveoli stage. *S. epidermidis* and *S. aureus* are responsible for colonization of critically ill patients, they may be the most important displayed nosocomial organisms, and their infection usually occurs after 48 and 72 h of admission. Also, *Alloiococcus otitidis*, *B. subtiles*, *B. thuringiensis*, *Kocuria rosea*, and *P. pseudoalcaligene*, reached the last stage (alveoli) in NICU#1. In NICU#2, eight species were identified in the last stage: *Bacillus cereus*, *Bacillus subtilis*, *Bacillus thuringiensis*, *Eikenella corrodens*, *Pseudomonas aeruginosa*, *Staphylococcus aureus*, *Staphylococcus epidermidis*, and *Streptococcus gordoni. S. cohnii* ssp. was only fixed in the trachea and bronchi stage in NICU#1. In contrast, *B. cereus* was fixed in all size ranges in both NICUs studied.

### 3.3. Antibiotic Resistance of Airborne Bacteria

Sepsis in the neonatal intensive care unit (NICU) remains one of the most significant causes of morbidity and mortality, especially for preterm newborns. Multi-drug-resistant organisms (MDROs) are emerging as important pathogens that cause neonatal sepsis in NICU [91]. Bacteria can be intrinsically resistant owing to its inherent properties or can achieve this resistance capacity via mutations and gene transfer. Various mechanisms of antibiotic resistance include poor penetration of drugs into the cell, an efflux of antibiotics by efflux pumps, target modification by mutation, and hydrolysis of antibiotics [92]. It had been reported that the increase in the episodes of sepsis due to MDROs at NICU had increased incidents were associated with high mortality, especially in preterm infants [91]. Thus, in our study, in order to assess antibiotic resistance in NICU#1 and NICU#2, 12 species and 8 species respectively, were evaluated according to the interpretation criteria currently suggested by the Standards Institute of the United States Clinical Laboratory (CLSI). The following levels or categories were used: susceptible (S), intermediate (I), and resistant (R). These categories are in relation to the minimum inhibitory concentration (MIC), which is interpreted as the concentration of an antimicrobial agent that inhibits bacterial growth [93]. 

In NICU#1, 100% of the strains of the species *Kocuria rosea*, *Bacillus thuringiensis*, *Alloiococcus otitis*, *Bacillus Megaterium*, *Bacillus pumilus*, and *Bacillus Subtiles* presented an intermediate susceptibility. This finding suggests that the antibiotic is unpredictable, given that the microorganisms present a minimum inhibitory concentration of the microbial agent very close to the levels of the antibiotic and for which the response will not be equal to those susceptible to the antibiotic [93]. For the eleven reported strains of *Bacillus cereus*, six reported resistance to *Ampicillin*, seven to *Cefoxitin*, five to *Ceftaroline*, and eight to *Penicillin G*. Four of these same strains showed resistance simultaneously to the five antibiotics, whereas to the other antibiotics they were susceptible in an intermediate way. Resistance to these antibiotics indicates that the probability of therapeutic success is nil or very reduced, therefore, antibiotics will not have any therapeutic effect. 

Strains *of Pseudomonas pseudoalcaligenes* were susceptible to Gentamicin, Amikacin, Cefepime Ceftazidime, Ciprofloxacin, Imipenem, Levofloxacin, Meropenem, Piperacillin-Tazobactam, and Trimetropin-Sulfamethoxazole. *Staphylococcus cohnii* ssp. *cohnii* was resistant in parallel to ampicillin and G penicillin and susceptible to Daptomycin, Linezolid, Vancomycin, Oxacillin, Minocycline, Rifampicin, Trimetropin-Sulfamethoxazole, Clindamycin, and Erythromycin. For *Staphylococcus epidermidis* a total of 95 strains were found, where some had resistance to Ampicillin (54 Strains), Penicillin G (59 Strains), and Clindamycin (59 Strains). Of these 59 resistant strains to any of the three antibiotics, 20 presented a simultaneous resistance to the three antibiotics. This microorganism also showed susceptibility to antibiotics such as Daptomycin, Linezolid, Vancomycin, Oxacillin, Minocycline, Rifampicin, Trimetropin-Sulfamethoxazole, and Erythromycin. Only one strain reported resistance and susceptibility to all of the mentioned antibiotics, for the other antibiotics *Staphylococcus epidermidis* is susceptible in an intermediate way. Gram-negative bacilli are increasing in incidence among nosocomial pathogens, largely because of their ability to express certain resistance phenotypes. Among those organisms, several species are very important causes of nosocomial infections because of their increasing resistance to the second- and third-generation cephalosporins and other extended-spectrum b-lactam agents [57].

In this research, 43 strains corresponding to *Staphylococcus saprophyticus* were identified, of which 23 strains were resistant to Ampicillin, 24 to Penicillin G, 6 to Oxaxilin, and 25 to Erythromycin. As observed with other microorganisms, *Staphylococcus saprophyticus* reported eight strains that showed resistance to the aforementioned antibiotics. Surprisingly, *S. saprophyticus* was prevalent in all size ranges (including alveoli), as well as resistant to several antibiotics. *K. rosea* presented a similar pattern. It is possible, therefore, that for controlling the dispersion of resistant airborne bacteria, airborne particles’ concentration must be reduced. The reason for this is not clear but it may have something to do with the fact that these particles may provide a rich environment with enough water and nutrients, therefore, giving enough time to exchange antibiotic resistant plasmids. Thus, increasing survival time for airborne bacteria. 

In NICU#2, 100% of *B. cereus* strains were identified as multi-drug-resistant organisms. The strains found were resistant to Ampicillin, Cefoxitin, Ceftaroline, and Penicillin G according to our results. *B. cereus* is usually susceptible to erythromycin, clindamycin, aminoglycosides, ciprofloxacin, vancomycin, and chloramphenicol [57].

*Pseudomonas aeruginosa* is located at the fourth position and constitutes the model of this category of aerobic nosocomial pathogens. It combines adaptability to a variety of moist environments with large potentialities of virulence factors. It is a pathogen of central importance in a broad range of nosocomial infections (and community-acquired infections) and it is the leading cause of nosocomial respiratory tract infections [94]. In this study, we have proven that the strains isolated are multi-resistant to the antibiotics Gentamicin, Amikacin, Cefepima, Ceftazidime, Ciprofloxacin, Levofloxacin, Meropenem, and Piperacillin-Tazobactam and finally that they are resistant to Imipenem. This coincides with reports by other authors about *P. aeruginosa* and its growing resistance to imipenem. 

Coagulase-negative staphylococci (CoNS) are the major cause of nosocomial bacteraemia in neonates [95]. Increasing incidences of resistant bacteria in Intensive Care Units is associated with poorer outcomes. These include methicillin-resistant Staphylococcus aureus (MRSA), and consequently, the strains of *Staphylococcus aureus* found in this study were resistant to Ampicilina and Penicilina G [96]. Finally, *Streptococcus gordoni* strains were resistant to Gentamicin, which is considered a colonizer microorganism. 

## 4. Conclusions

Public-style NICU revealed higher concentrations of airborne viable bacteria (53.00 to 214.37 CFU/m^3^) than private-style NICU (151.94–466.43), indicating a possible threat to health. Results indicated no statistically significant difference between concentrations before and after visits, as well as before and after cleaning activities. 

Our research identified some of the species which are considered as aggressive pathogens and are highly dangerous for the health of the newborns, and are considered capable of causing respiratory infections, cases of pneumonia, and infections of the skin and eyes. Other species founded in this study are recognized as opportunist, a normal bacterium of human skin. However, these microorganisms can also emerge as an intrahospital pathogen that infects immunocompromised patients, such as newborns. Infections related to the colonization of foreign bodies (intravenous catheters, hemodialysis fistulas, pacemakers, prosthetic joints, valvular grafts and/or prosthetic heart valves) may eventually produce urinary tract infections, bacteremia, and sepsis. Other species identified generate biofilms which facilitate pathogenic permanence. 

The findings of this study have a number of important implications for future practice. Special efforts must be warranted to reduce airborne contaminants in NICUs. The Colombian National requirements of the unique system of qualification of the Health System do not provide specific information about the air conditioning maintenance or protocols to ensure the monitoring of bioaerosols, neither does it improve the indoor air quality. Therefore, it is important to consider the use of air purification systems, possibly involving overnight UV irradiation, including protocols for reducing particle matter. 

Our findings may help to understand the concentration of airborne viable bacteria in different size ranges, as well as their antibiotic resistance in a NICU. Moreover, our results may indicate a potential health threat to humans due to the antibiotic resistance of airborne bacteria in the NICU, considered to be pathogens or pathogenic under certain circumstances. These data must be interpreted with caution because of the complicated correlations between airborne bacteria and environmental factors. This is an important issue for future research, thus more studies are needed to provide proper exposure limits in relation to health in NICU indoor environments.

## Figures and Tables

**Table 1:** Comparison of the concentration levels of bioaerosols with governmental and private organizations guidelines (adapted from [3]).

Organization	Value		Reference
Concentration of bacteria in air (mean and range)	110.13 CFU/m^3^ (53.00–214.37 CFU/m^3^)	-	This study—NICU#1
	310.37 CFU/m^3^ (151.94–466.43 CFU/m^3^)	-	This study—NICU#2
American Conference of Governmental Industrial Hygienists (ACGIH)	<100 CFU/m^3^	Low	[34]
	100–1000 CFU/m^3^	Intermediate	
	>1000 CFU/m^3^	High.	
Healthy Buildings International	<750 CFU/m^3^	The total of bacteria and fungi in the air are fine if the species are not infectious or allergenic.	[35]
Indoor Air Quality Association (IAQ)	<150 CFU/m^3^	If it is a mixture of species it is fine.	[36]
The Netherlands/research methods in biological indoor air pollution	>104 CFU/m^3^	The total fungus and bacteria are a threat to health.	[37]
	>500 CFU/m^3^	A species of potentially pathogenic nature is a threat to health.	
Occupational Safety and Health Administration (OSHA)	>1000 CFU/m^3^	Indicates contamination	[38]
	>106 Fungus/Bacteria/dust	Indicates contamination	

**Table 2:** ANOVA for bacteria bioaerosols concentration (CFU/m^3^) between the groups, for NICU#1 and NICU#2.

NICU#1—Airborne Bacteria					
Source	Sum of Squares	*df*	Mean Square	F-Value	*p*-Value
Between groups	58.82	1	58.82	0.22	0.639
Intra groups	5.18 × 10^4^	194	267.20	-	-
Total (Corrected.)	5.19 × 10^4^	195	-	-	-
NICU#2—airborne bacteria					
Between ** groups	1300.64	1	1300.64	0.06	0.81
Intra groups	81,217.98	4	20,304.49	-	-
Total (Corrected.)	82,518.61	5	-	-	-
NICU#2—particle number					
Between ** groups	15,116,795.93	1.00	15,116,795.93	0.08	0.79
Intra groups	1,971,755,605.47	10.00	197,175,560.55	-	-
Total (Corrected.)	1,986,872,401	11	-	-	-

* Groups: before (1) and after (2) visits. ** Groups: before (1) and after (2) cleaning.

**Table 3:** Mean concentration of bacterial bioaerosols per campaign before and after the visit time.

Microorganism	Before (CFU/m^3^)					After (CFU/m^3^)					Total
	1	2	3	4	5	1	2	3	4	5	
*Alloiococcus otitidis*		7			7			7			7
*Bacillus cereus*	7	7	**21**		7	7	7	7		7	**8**
*Bacillus megaterium*	7		7	7	7	7		9			**8**
*Bacillus pumilus*			7		7				7	7	7
*Bacillus subtilis*	7		7				7			7	7
*Bacillus thuringiensis*	7		7			7		7			7
*Kocuria. Rosea*	7		9	7	7			**28**	**28**		**12**
*Leifsonia aquatica*	7		7		7						7
*Pseudomona pseudoalcaligenes*			7				7	7			7
*S. cohnii* ssp.	7										7
*S. epidermidis*	11	**13**	**27**	**18**	**11**	8	**15**	**20**	**16**	**19**	**16**
*S. saprophyticus*	**33**	7	**21**		7	**18**	9	**30**	7		**21**

Note: Highest value in bold.

**Table 4:** Mean concentration of bacterial bioaerosols per campaign before and after cleaning time NICU#2.

Microorganism	Before (CFU/m^3^)			After (CFU/m^3^)			Total
	1	2	3	1	2	3	
*Bacillus cereus*			**13**			**25**	**38**
*Bacillus subtilis*	**10**	**7**	**24**	**12**		**22**	**77**
*Bacillus thuringiensis*			**14**			**13**	**27**
*Eikenella corrodens*		**12**			**16**		**28**
*Pseudomonas aeruginosa*		**10**			**11**		**20**
*Staphylococcus aureus*	**7**			**12**			**19**
*Staphylococcus epidermidis*	**10**			**15**			**25**
*Streptococcus gordoni*	**13**			**12**			**26**

Note: Highest value in bold.

## Supplementary Materials

The following are available online at https://www.mdpi.com/1660-4601/16/18/3340/s1, Figure S1: Means and 95.0% LSD intervals concentration #/L particle by species for NICU#2. Figure S2: Means and 95.0% LSD intervals particle size by species for NICU#2. Figure S3: Means and 95.0% LSD intervals Concentration #L by stages of cascade impactor for NICU#2. Figure S4: Fitted model with 95.0% confidence limits Cefoxitin in NICU#2. Figure S5: Fitted model with 95.0% confidence limits Gentamicin in NICU#2. Table S1: Statistic summary for concentration by species for NICU#1. Table S2: Table S2. Statistic summary for concentration by stages for NICU#1. Table S3: Statistic summary concentration #/L particle by species for NICU#2. Table S4: Summary Statistics for particle size by species. Table S5: Simple regression CFU/m3 vs. Concentration #L NICU#2. Table S6: Simple regression CFU/m3 vs. Concentration #L NICU#2. Table S7: Summary statistics for Concentration #L by stages of cascade impactor NICU#2. Table S8: Logistic regression for cefoxitin NICU#2. Table S9: Logistic regression for Gentamicin NICU#2. Table S10: Data antibiotic resistance and susceptible airborne bacteria in NICU#1 and NICU#2.
