# Supplementary material for: Antibiotic Resistance of Airborne Viable Bacteria and Size Distribution in Neonatal Intensive Care Units
Source: Int J Environ Res Public Health. 2019 Sep 10;16(18):3340. doi: 10.3390/ijerph16183340 (PMC6765827; doi:10.3390/ijerph16183340)
Supplement: Supplementary file 1 [file ijerph-16-03340-s001.zip › ijerph-483375-supplementary v2.docx]

Article

Antibiotic Resistance of Airborne Viable Bacteria and Size Distribution in Neonatal Intensive Care Units

Wendy Beatriz Morgado-Gamero ^1,^*, Martha Mendoza Hernandez ^2^, Margarita Castillo Ramirez ^3^, Jhorma Medina ^1^, Stephanie De La Hoz ^1^, Heidy Posso Mendoza ^4^, Alexander Parody ^5^, Elba C. Teixeira ^6^, and Dayana Milena Agudelo-Castañeda ^2,^*

^1^ Department of Exact and Natural Sciences, Universidad de la Costa, Calle 58#55-66, Barranquilla 080002, Colombia; jhorma96@hotmail.com (J.M.); sdelahoz12@outlook.com (S.D.L.H.)

^2^ Department of Civil and Environmental Engineering, Universidad del Norte, Km 5 Vía Puerto Colombia, Barranquilla: 081007. Colombia; Marticamh15@hotmail.com

^3^ Barranquilla Air Quality Monitoring Network. EPA—Barranquilla Verde, Barranquilla Post code:080001, Colombia; mcastilloramirez87@gmail.com (M.C.R.)

^4^ Department of Bacteriology, Universidad Metropolitana, Calle 76 No. 42-78, Barranquilla 080020, Colombia; Heidy_posso@unimetro.edu.co

^5^ Engineering Faculty, Universidad Libre Barranquilla, Carrera 46 No. 48-170, Barranquilla 080002, Colombia; alexandere.parodym@unilibre.edu.co

^6^ Postgraduate Program in Remote Sensing. Universidade Federal do Rio Grande do Sul, Av. Bento Gonçalves, 9500, Porto Alegre, RS 91501-970, Brazil; ecalessoteixeira@gmail.com

***** Correspondence: wmorgado1@cuc.edu.co (W.M.-G.); D.A.C: mdagudelo@uninorte.edu.co (D.M.A.-C.); Tel.: +57-5-350 9509 EXT 3896 (D.M.-A.-C.)

Received: 28 March 2019; Accepted: 16 May 2019; Published: date

**Supplementary Information**

**Table S1.** Statistic summary for concentration by species for NICU #1.

| **Species** | **Statistic** | **Before** | **After** |
| --- | --- | --- | --- |
| *K. rosea* | Average | 7.95053 | 28.26855 |
|  | Median | 7.067138 | 28.26855 |
|  | Standard Deviation | 2.498611 | 0 |
| *Bacillus Cereus* | Average | 9.42285 | 7.067138 |
|  | Median | 7.067138 | 7.067138 |
|  | Standard Deviation | 5.770294 | 0 |
| *Staphylococcus epidermidis* | Average | 15.68905 | 16.22229 |
|  | Median | 7.067138 | 14.13428 |
|  | Standard Deviation | 14.85781 | 11.15639 |
| *Bacillus megaterium* | Average | 7.067138 | 8.833922 |
|  | Median | 7.067138 | 7.067138 |
|  | Standard Deviation | 0 | 3.533569 |
| *Staphylococcus saprophyticus* | Average | 22.68923 | 20.02356 |
|  | Median | 14.13428 | 14.13428 |
|  | Standard Deviation | 20.47966 | 31.30606 |

**Table S2.** Statistic summary for concentration by stages for NICU #1.

| **Species** | **Statistic** | **Before** | **After** |
| --- | --- | --- | --- |
| > 7.0 μm (stage 1). | Average | 16.25442 | 13.82701 |
|  | Median | 10.60071 | 7.067138 |
|  | Standard Deviation | 17.62688 | 10.32477 |
| 4.7–7.0 μm (stage 2) | Average | 9.42285 | 7.067138 |
|  | Median | 7.067138 | 7.067138 |
|  | Standard Deviation | 5.770294 | 0 |
| 3.3–4.7 μm (stage 3) | Average | 12.36749 | 18.8457 |
|  | Median | 7.067138 | 14.13428 |
|  | Standard Deviation | 9.135633 | 12.90277 |
| 2.1–3.3 μm (stage 4) | Average | 11.18963 | 14.77674 |
|  | Median | 7.067138 | 14.13428 |
|  | Standard Deviation | 10.20053 | 8.029553 |
| 1.1–2.1 μm (stage 5) | Average | 21.20141 | 20.52835 |
|  | Median | 7.067138 | 7.067138 |
|  | Standard Deviation | 21.6386 | 33.66397 |
| 0.65–1.1 μm (stage 6) | Average | 12.11509 | 9.42285 |
|  | Median | 7.067138 | 7.067138 |
|  | Standard Deviation | 15.03736 | 5.770294 |

**Table S3.** Statistic summary concentration #/L particle by species for NICU #2.

**Summary Statistics for Concentration #/L**

| *species* | *Count* | *Average* | *Standard deviation* | *Minimum* | *Maximum* |
| --- | --- | --- | --- | --- | --- |
| Bacillus Cereus | 12 | 7801.974 | 15,609.43 | 42.88 | 45,893.21 |
| Bacillus Subtilis | 24 | 6220.137 | 13,758.8 | 42.88 | 45,893.21 |
| Bacillus Thuringiensis | 10 | 9341.042 | 16,793.09 | 42.88 | 45,893.21 |
| Eikenella Corrodens | 8 | 7029.256 | 11,543.52 | 45.5 | 31,214.49 |
| Pseudomonas Aeruginosa | 10 | 5722.591 | 1,0548.35 | 45.5 | 31,214.49 |
| Shigella Dysenteriae | 2 | 9168.995 | 12,869.46 | 68.91 | 18,269.08 |
| Staphylococcus Aerous | 5 | 15,459.38 | 21,675.39 | 45.17 | 45,824.41 |
| Staphylococcus epidermidis | 10 | 8697.93 | 16,162.21 | 45.17 | 45,824.41 |
| Streptococcus Gordoni | 10 | 8242.552 | 16,366.35 | 45.17 | 45,824.41 |
| Total | 91 | 7855.133 | 14,434.3 | 42.88 | 45,893.21 |

**ANOVA Table for Concentration #/L by species**

| *Source* | *Sum of Squares* | *Df* | *Mean Square* | *F-Ratio* | *P-Value* |
| --- | --- | --- | --- | --- | --- |
| Between groups | 4.383835 × 10^8^ | 8 | 5.479793 × 10^7^ | 0.25 | 0.9807 |
| Within groups | 1.831304 × 10^10^ | 82 | 2.233297 × 10^8^ |  |  |
| Total (Corr.) | 1.875142 × 10^10^ | 90 |  |  |  |

**Figure S1.** Means and 95.0 % LSD intervals concentration #/L particle by species for NICU #2.

**Table S4.** Summary Statistics for particle size by species

**Summary Statistics for particle size**

| *species* | *Count* | *Average* | *Standard deviation* | *Minimum* | *Maximum* |
| --- | --- | --- | --- | --- | --- |
| Bacillus Cereus | 12 | 3.216667 | 3.58046 | 0.3 | 10.0 |
| Bacillus Subtilis | 24 | 3.5375 | 3.75289 | 0.3 | 10.0 |
| Bacillus Thuringiensis | 10 | 3.11 | 3.904542 | 0.3 | 10.0 |
| Eikenella Corrodens | 8 | 3.45 | 4.33359 | 0.3 | 10.0 |
| Pseudomonas Aeruginosa | 10 | 3.36 | 3.941009 | 0.3 | 10.0 |
| Shigella Dysenteriae | 2 | 2.65 | 3.323402 | 0.3 | 5.0 |
| Staphylococcus Aerous | 5 | 4.62 | 4.992695 | 0.3 | 10.0 |
| Staphylococcus epidermidis | 10 | 3.11 | 3.904542 | 0.3 | 10.0 |
| Streptococcus Gordoni | 10 | 3.71 | 3.745946 | 0.3 | 10.0 |
| Total | 91 | 3.432967 | 3.741154 | 0.3 | 10.0 |

**ANOVA Table for particle size by species**

| *Source* | *Sum of Squares* | *Df* | *Mean Square* | *F-Ratio* | *P-Value* |
| --- | --- | --- | --- | --- | --- |
| Between groups | 12.00418 | 8 | 1.500523 | 0.10 | 0.9992 |
| Within groups | 1247.657 | 82 | 15.21533 |  |  |
| Total (Corr.) | 1259.661 | 90 |  |  |  |

**Figure S2.** Means and 95.0 % LSD intervals particle size by species for NICU #2.

**Table S5.** Simple regression CFU/m^3^ vs Concentration #L NICU #2.

**Simple Regression - CFU/m3 vs. Concentration #/L**

Dependent variable: CFU/m3

Independent variable: Concentration #/L

Multiplicative model: Y = a*X^b

Number of observations: 91

**Coefficients**

|  | *Least Squares* | *Standard* | *T* |  |
| --- | --- | --- | --- | --- |
| *Parameter* | *Estimate* | *Error* | *Statistic* | *P-Value* |
| Intercept | 5.409349 | 0.1249756 | 43.28324 | 0.0000 |
| Slope | 0.01666633 | 0.01782292 | 0.9351066 | 0.3523 |

NOTE: intercept = ln(a)

**Analysis of Variance**

| *Source* | *Sum of Squares* | *Df* | *Mean Square* | *F-Ratio* | *P-Value* |
| --- | --- | --- | --- | --- | --- |
| Model | 0.1548469 | 1 | 0.1548469 | 0.87 | 0.3523 |
| Residual | 15.76051 | 89 | 0.1770844 |  |  |
| Total (Corr.) | 15.91536 | 90 |  |  |  |

**Table S6.** Simple regression CFU/m^3^ vs Concentration #L NICU #2.

**Simple Regression - CFU/m3 vs. particle size**

Dependent variable: CFU/m3

Independent variable: particle size

Reciprocal-Y logarithmic-X model: Y = 1/(a + b*ln(X))

Number of observations: 91

**Coefficients**

|  | *Least Squares* | *Standard* | *T* |  |
| --- | --- | --- | --- | --- |
| *Parameter* | *Estimate* | *Error* | *Statistic* | *P-Value* |
| Intercept | 0.004241681 | 0.0001470933 | 28.83666 | 0.0000 |
| Slope | 0.0001191272 | 0.0001048274 | 1.136413 | 0.2588 |

**Analysis of Variance**

| *Source* | *Sum of Squares* | *Df* | *Mean Square* | *F-Ratio* | *P-Value* |
| --- | --- | --- | --- | --- | --- |
| Model | 0.000002258622 | 1 | 0.000002258622 | 1.29 | 0.2588 |
| Residual | 0.0001556545 | 89 | 0.000001748927 |  |  |
| Total (Corr.) | 0.0001579131 | 90 |  |  |  |

**Table S7.** Summary statistics for Concentration #L by stages of cascade impactor NICU #2.

**Summary Statistics for Concentration #/L**

| *Stage* | *Count* | *Average* | *Standard deviation* | *Minimum* | *Maximum* |
| --- | --- | --- | --- | --- | --- |
| E1 | 19 | 45.97105 | 2.192796 | 42.88 | 48.57 |
| E2 | 14 | 71.93357 | 5.573319 | 66.96 | 81.16 |
| E3 | 10 | 163.092 | 17.34189 | 145.71 | 192.24 |
| E4 | 14 | 785.6786 | 115.7052 | 562.49 | 910.7 |
| E5 | 16 | 4156.722 | 972.2285 | 2154.11 | 5205.77 |
| E6 | 18 | 35,211.03 | 10,145.62 | 18,269.08 | 45,893.21 |
| Total | 91 | 7855.133 | 14,434.3 | 42.88 | 45,893.21 |

**ANOVA Table for Concentration #/L by Stage**

| *Source* | *Sum of Squares* | *Df* | *Mean Square* | *F-Ratio* | *P-Value* |
| --- | --- | --- | --- | --- | --- |
| Between groups | 1.698719 × 10^10^ | 5 | 3.397439 × 10^9^ | 163.69 | 0.0000 |
| Within groups | 1.764227 × 10^9^ | 85 | 2.075562 × 10^7^ |  |  |
| Total (Corr.) | 1.875142 × 10^10^ | 90 |  |  |  |

**Figure 3.** Means and 95.0 % LSD intervals Concentration #L by stages of cascade impactor for NICU #2.

**Table S8.** Logistic regression for cefoxitin NICU #2.

**Logistic Regression - Cefoxitina**

Dependent variable: Cefoxitina

Factors:

Temperature

Concentration #/L

particle size

Number of observations: 17

**Estimated Regression Model (Maximum Likelihood)**

|  |  | *Standard* | *Estimated* |
| --- | --- | --- | --- |
| *Parameter* | *Estimate* | *Error* | *Odds Ratio* |
| CONSTANT | -1606.426 | 4878.778 |  |
| Temperature | 67.92499 | 202.5079 | 3.158249 × 10^29^ |
| Concentration #/L | 0.0 | 0.005714882 | 1.0 |
| particle size | 0.0 | 24.87167 | 1.0 |

**Analysis of Deviance**

| *Source* | *Deviance* | *Df* | *P-Value* |
| --- | --- | --- | --- |
| Model | 20.59711 | 3 | 0.0001 |
| Residual | 1.804334 × 10^−12^ | 13 | 1.0000 |
| Total (corr.) | 20.59711 | 16 |  |

Percentage of deviance explained by model = 100.0

Adjusted percentage = 61.15961

**Likelihood Ratio Tests**

| *Factor* | *Chi-Square* | *Df* | *P-Value* |
| --- | --- | --- | --- |
| Temperature | 17.97581 | 1 | 0.0000 |
| Concentration #/L | 0.0 | 1 | 1.0000 |
| particle size | 0.0 | 1 | 1.0000 |

**Figure S4.** Fitted model with 95.0% confidence limits Cefoxitin in NICU #2.

**Table S9.** Logistic regression for Gentamicin NICU #2.

**Logistic Regression - Gentamicina**

Dependent variable: Gentamicina

Factors:

Temperature

Concentration #/L

particle size

Number of observations: 20

**Estimated Regression Model (Maximum Likelihood)**

|  |  | *Standard* | *Estimated* |
| --- | --- | --- | --- |
| *Parameter* | *Estimate* | *Error* | *Odds Ratio* |
| CONSTANT | 777.0261 | 1834.064 |  |
| Temperature | -32.17499 | 75.19203 | 1.06311 × 10^−14^ |
| Concentration #/L | 0.0 | 0.00613588 | 1.0 |
| particle size | 0.0 | 21.97298 | 1.0 |

**Analysis of Deviance**

| *Source* | *Deviance* | *Df* | *P-Value* |
| --- | --- | --- | --- |
| Model | 27.72589 | 3 | 0.0000 |
| Residual | 2.122746 × 10^−12^ | 16 | 1.0000 |
| Total (corr.) | 27.72589 | 19 |  |

Percentage of deviance explained by model = 100.0

Adjusted percentage = 71.1461

**Likelihood Ratio Tests**

| *Factor* | *Chi-Square* | *Df* | *P-Value* |
| --- | --- | --- | --- |
| Temperature | 27.32063 | 1 | 0.0000 |
| Concentration #/L | 0.0 | 1 | 1.0000 |
| particle size | 0.0 | 1 | 1.0000 |

**Figure S5.** Fitted model with 95.0% confidence limits Gentamicin in NICU #2.

**Table S10.** Data antibiotic resistance and susceptible airborne bacteria in NICU #1 and NICU #2. (see Excel)
